# Impact of a PMMA tube on performances of a Vereos PET/CT system adapted for BSL-3 environment according to the NEMA NU2-2012 standard

**DOI:** 10.1186/s40658-022-00450-6

**Published:** 2022-03-22

**Authors:** Nidhal Kahlaoui, Thibaut Naninck, Roger Le Grand, Catherine Chapon

**Affiliations:** Inserm, CEA, Center for Immunology of Viral, Auto-immune, Hematological and Bacterial Diseases (IMVA-HB/IDMIT), Fontenay-aux-Roses & Le Kremlin-Bicêtre, Université Paris-Saclay, Paris, France

**Keywords:** PET/CT, NEMA NU2-2012 standard, BSL-3 conditions, Infectious diseases

## Abstract

**Introduction:**

A Vereos PET/CT device was adapted to be compatible with the experimentation in large animals within BSL-3 environment. The aim of this study was to investigate the impact of this modification on the performance according to NEMA NU2-2012 standard.

**Methods:**

Spatial resolution, sensitivity, count rate performance, accuracies of corrections and image quality were assessed using the NEMA NU2-2012 standards before and after installation of a transparent poly-methyl methacrylate tube of 8 mm thickness, 680 mm diameter and 2800 mm long inside the tunnel of the system. In addition, CT performance tests were performed according to manufacturer standard procedure.

**Results:**

Although the presence of the tube led to a slight decrease in sensitivity, performance measurements were in accordance with manufacturer preconisation ranges and comparable to previous performance published data.

**Conclusion:**

Modifications of Vereos PET/CT system allowing its use in BSL-3 conditions did not affect significantly its performance according to NEMA NU2-2012 standard.

**Key points:**

*Question.* Does a BSL-3 compatible modification alter Philips Vereos PET/CT performances according to NEMA NU2-2012 standards?

*Pertinent findings.* Our Vereos PET/CT system was modified by a wall separating BSL-1 and BSL-3 sides and an 8 mm thickness PMMA tube inserted into the bore of the camera in order to extend the BSL-3 containment along the bed movement. The performances of our modified system according to NEMA NU2-2012 standards were not significantly impacted by the modifications and were in accordance with the values prescribed by the manufacturer.

*Implications for patients care.* Our clinical PET/CT device was modified for human infectious diseases studies in Non-Human Primates. This unusual set up may then provide truly transposable data from preclinical studies into clinical application in infected patients.

## Introduction

Preclinical and clinical PET/CT imaging have extensively been used for evaluating diseases and drug treatment in neurology, oncology or cardiology [1, 2]. The current pandemic of COVID-19 is an illustration of the crucial need for specific tools for a better understanding of human infectious diseases, in order to develop and improve prevention strategies and therapies. However, the manipulation of infectious pathogens requires specific installations in research facilities especially for imaging, such as PET [3, 4]. Biosafety levels, depending on the infectivity and transmissibility of the pathogenic agents and on the conducted work, are designated as basic (BSL-1, BSL-2), containment (BSL-3) and maximum containment (BSL-4) [5]. They dictate the type of work practices that are allowed to occur in a lab setting and play a huge role in the design of the facility [6]. The application of imaging technologies including PET/CT in BioSafety Level 3 or 4 (BSL-3 or 4) has to face challenges in term of safety, cross-contaminations and maintenance of equipment in this environment [7, 8]. Actually, BSL-3 or 4 laboratories are access restricted, air tight areas with extended decontamination and waste control procedures [9]. Different strategies were used to overcome these issues including the installation of imaging device in BSL-1 environment with the use of isolation sealed chambers for the infected animals to avoid contamination [10, 11]. Specific procedures for the decontamination of installations were also developed for imaging devices installed directly in BSL-3 environment, although this approach could be damaging for the equipment. Another approach was to separate the imaging suite into two sides: a biologically “hot” side (BSL-2/3/4) for animal preparation and imaging, and a biologically “cold” side (BSL-1) with the PET/CT gantry, where the hardware maintenance would have been facilitated [12]. We adapted our PET/CT device (Vereos, Philips Healthcare) in a similar manner to be compatible with the experimentation in large animals within BSL-3 environment, with the introduction of a transparent poly-methyl methacrylate (PMMA) biocontainement tube into the bore of the imaging system on the cold side for extending the BSL-3 side. However, to our knowledge, we did not found any study describing the alterations of a clinical PET/CT device performances with such a tube insert. The aim of this study was then to investigate the impact of this modification on the performance of the Vereos-Ingenuity PET/CT device according to NEMA NU2-2012 standard.

## Material and methods

### PET/CT facilities

**Fig. 1:**
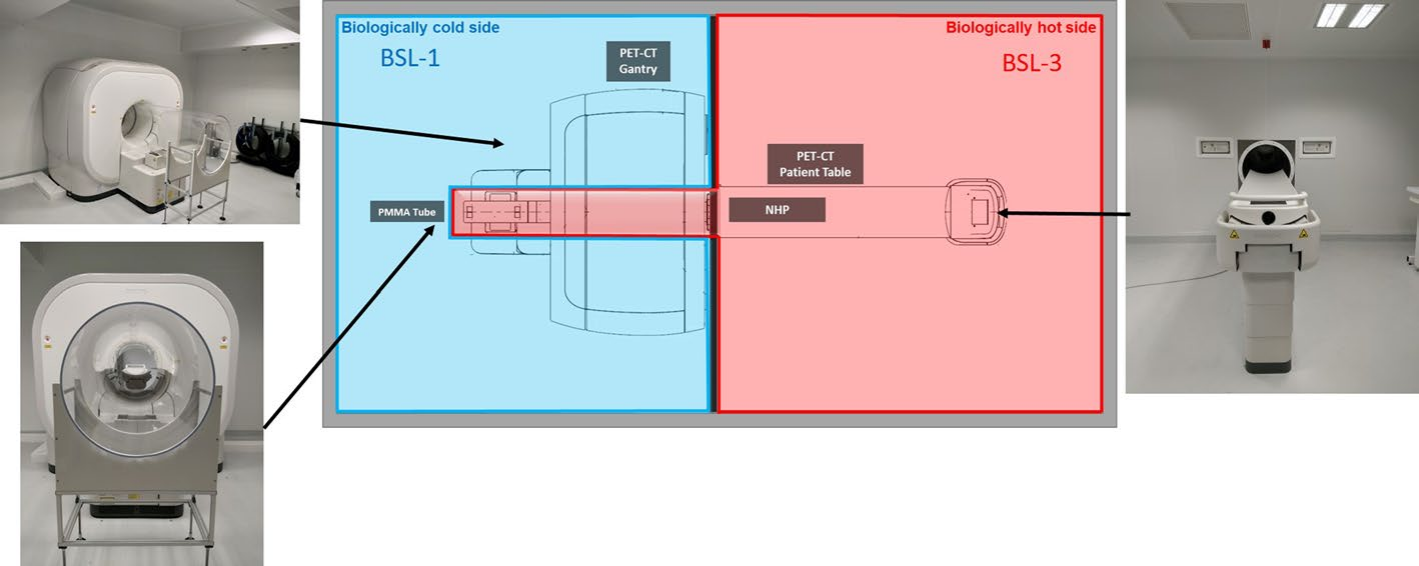
PET/CT (Vereos, Philips Healthcare) imaging facilities allowing for imaging to be performed in BSL-2 and BSL-3 environment by separating the BSL-1 side (in blue) with the PET/CT device, from the BSL-3 (in red) with the Non-Human Primate (NHP) preparation room. A transparent poly-methyl methacrylate (PMMA) containment tube (2800 mm of length, 680 mm of diameter and 8 mm thickness) extends the partition between BSL-1 and BSL-3 sides into the PET/CT gantry, also extending the biological barrier into imaging system

The device studied here is the Vereos digital PET/CT system (Philips Healthcare, The Netherlands). As already reported [13, 14], the main characteristic of the Vereos system relies on its detector technology using digital photon counting (DPC) instead of classical photomultipliers (PMT). In order to use the Vereos system for infectious diseases research and BioSafety Level 3 pathogen studies, a specific set up was designed where the Vereos PET/CT gantry device was physically isolated from the BSL-3 room. As described in Fig. 1, the PET/CT imaging facility was divided into two separate rooms, a “biologically hot room”, BSL-3, where pathogens are present (in red) and a “biologically cold room”, BSL-1 (in blue). The PET/CT gantry was located in the “biologically cold room” and the machine bed in the BSL-3 room. In order to prolong the BSL-3 containment along the machine bed movement across the gantry, a tube, closed at its extremity, was inserted in the channel of the gantry and fixed to the wall separating the two rooms (Fig. 1). The tube, produced by JCE biotechnology (Vichy, France), was made of transparent poly-methyl methacrylate (PMMA) and measured 2800 mm of length, 680 mm of diameter and 8 mm thickness. Before all NEMA PET measurements, energy and timing measurements were performed according to manufacturer daily QC recommendations with a 15.9 MBq ^22^Na point source (Epsilon Radioactive Sources).

### CT acceptance test

CT acceptance tests were performed according to manufacturer standard procedure to assess noise levels. These tests were performed before and after PET/CT device modification in order to estimate the effect of the PMMA tube on CT image quality and noise.

Head and body noises were measured using a Philips Quality Assurance CT phantom (Extended Phantom Kit, Philips Medical Systems, USA) without and with tube samples of 8 and 10 mm thickness. Head acceptance test was performed using a 120 kV, 200mAs, 64 × 0.625 mm CT acquisition in a 250 mm field of view (FOV). Body CT acceptance test settings were 120 kV voltage, 400mAs intensity and 32 × 1.25 collimation in a 350 mm FOV. In both head and body parts, noise measures were performed in 8 different images. Acceptable noise levels were set by the manufacturer for both head (4.4–6) and body (11.5–15.5) parts of the phantom.

### National Electrical Manufacturers Association (NEMA) NU2-2012 measurements

Initial measurements on the PET/CT system were performed before tube installation between January and February 2019. The second measures were performed between May and July 2019, following the installation of the 8 mm thickness PMMA biocontainment tube within the bore of the Vereos PET/CT system. All phantoms (NEMA PET sensitivity phantom, NEMA PET scatter phantom and NEMA International Electrotechnical Commission body phantom) used were purchased from Data Spectrum Corporation (USA).

For all PET measurements, the transaxial FOV was set to 576 mm and the default detection parameters (energy window of 450–613 keV and coincidence cutoff of 4 ns) were used. According to NEMA NU2-2012 protocols, spatial resolution, sensitivity, count-rate performance, accuracies of corrections, and image quality were assessed as described below. All reconstructions parameters were set according NEMA manufacturers guidelines and were identical to previous publications describing NEMA performances of a non-modified Vereos system [13].

### Spatial resolution

Spatial resolution was assessed using a ^18^F point source (axial diameter < 1 mm) introduced into a 100 mm capillary tube (Pyrex capillary tubes, catalog number 9530–1; Corning Life Science). This point source was about 3 mm long in the capillary and is composed of a droplet from a 2.0 GBq/mL ^18^F solution (PETNET solutions, Siemens Healthcare, France). In addition of the center of the FOV, transverse (radial and tangential) and axial positions were made at 1, 10, and 20 cm shifted from the FOV center as described in the NEMA NU2-2012 spatial resolution protocol.

Acquisition for each position and image reconstructions were performed according to manufacturer protocol [13]. Full width at half maximum (FWHM) values for center, axial and transverse (radial, tangential) positions were then assessed and compared with the limits values given by the manufacturer.

### Sensitivity, count rate performance and accuracies of corrections

Sensitivity measurements and count rate performance were evaluated using the NEMA PET Sensitivity and Scatter Phantoms respectively (Data Spectrum Corporation, 0118-002).

The NEMA PET Sensitivity phantom is composed of 5 different concentric sections of aluminum sleeves of 70-cm length each, with one 80-cm of a polyethylene tube at the center, filled with an ^18^F aqueous solution. Following the process given by the manufacturer, a 7.5 MBq ^18^F solution was prepared and injected into the polyethylene tube. For each aluminum sleeve added, a 2 min acquisition measurement was made for two radial positions (0 and 10 cm). For the both radial positions, sensitivity results obtained were expressed as the rate in counts per second by a given injected activity.

Count rate performance included a count of total, random and true events with the scatter fraction in addition of the noise equivalent count rate (NECR). The NEMA PET scatter phantom, similar to the NEMA PET Sensitivity phantom with the polyethylene tube containing a ^18^F aqueous solution of 1.15 GBq, was placed on the Vereos PET/CT patient table on the lower position as described in NEMA NU2-2012 protocols. The acquisitions (about 15 h long) and data analysis were performed according to the NEMA NU2-2012 standard manufacturer protocols resulting in 35 slices (5 mm spacing). A dedicated manufacturer reconstruction protocol called NEMA-Count-Loss was used to assess the accuracy of count losses and random corrections by extracting the relative count rate error. For each activity concentration, we extracted the relative count rate error in percentage units and calculated the highest, lowest and average values among these slices as previously described [13].

### Image quality

For the Image Quality NEMA test, both NEMA IEC Body and NEMA PET Scatter Phantoms (Data Spectrum Corporation) were used. The NEMA IEC Body phantom is composed to several fillable compartments with 6 spheres (10, 13, 17, 22, 28, and 37 mm of diameter), a lung insert and a background zone. The NEMA IEC Body Phantom was filled with aqueous ^18^F solutions with an activity of 4 times the background concentration as described in NEMA NU2-2012 protocols. The background compartment was first filled to a quarter with water and a 55 MBq ^18^F solution, resulting to a 22.7 kBq/mL initial radioactive solution. This solution was then used to fill the 10, 13, 17 and 22 mm diameter so-called ‘hot spheres’ with the same activity concentration. The two other spheres (28 and 37 mm of diameter), named ‘cold spheres’, were filled with water. After filling the rest of the background with water (resulting to a 5.67 kBq/mL background activity concentration), the scatter phantom was filled as already described in count rate performance section. The IEC Body Phantom was placed 30.5 cm from the front edge of the Vereos PET/CT patient table and the scatter phantom was placed at the end of the IEC Body Phantom. The image quality NEMA test was performed by a 3 min PET acquisition and a low dose CT scan. The Contrast and Variability percentages were then assessed for the six spheres and the relative error in the lung insert using the NEMA NU2-2012 standard manufacturer protocols. The images acquired in the phantom experiments were evaluated using different indicators [13, 15]: Phantom Noise Equivalent Count (NEC), the percentage contrast for spheres with different diameters, the percentage background variability associated and the average lung residual.

## Results

### CT acceptance test

Tube insertion in the PET/CT gantry increased the noise with an effect of the PMMA thickness (Table 1). The 10 mm thickness PMMA tube increased the head and body noise (6.15 HU and 16.2 HU respectively) by 24% and 23% respectively compared to noise values without tube (4.96 HU and 13.15 HU). These data were out of manufacturer acceptable range. A second noise measurement was then performed with an 8 mm thickness PMMA tube and provided better mean noise values of 5.61 HU and 14.9 HU respectively (a 13% increase of head and body noise compared to non-modified set up). This tube thickness then induced acceptable noise levels and was then chosen to modify our PET/CT device to be compatible with experimentation in BSL-3 environment.

**Table 1:** CT acceptance test without and with tube samples of 10 and 8 mm thickness

Parameter	Without tube	With 10 mm tube	With 8 mm tube	Acceptable range
CT acceptance test				
Head noise	4.96 ± 0.09	6.15 ± 0.13	5.61 ± 0.08	[4.4,6]
Body noise	13.15 ± 0.29	16.24 ± 0.27	14.91 ± 0.38	[11.5,15.5]

### Energy, timing and spatial resolution

The measured energy resolution was 11.00% FWHM and the timing resolution 311.8 ps FWHM without tube. These data were not impacted by the 8 mm PMMA tube (11.03% and 312.0 ps respectively, Table 2). In the same way, the presence of the tube did not modify the spatial resolution whatever the transverse and axial positions (Table 2).

**Table 2:** Performance parameters of the PET component of the PET/CT Vereos with or without an 8 mm PMMA biocontainment tube within the bore defined by NEMA NU2-2012 measurements including energy and timing resolution, spatial resolution, sensitivity, count rates and scatter fractions

Parameter	Without tube	With 8 mm tube	Manufacturer guidelines
Energy resolution (%)	11	11.03	≤ 13
Timing resolution (ps)	311.82	312	≤ 390
Spatial resolution (FWHM)			
Transverse, 1 cm	4.03 ± 0.11	3.96 ± 0.14	< 4.9
Transverse, radial, 10 cm	4.59 ± 0.09	4.62 ± 0.09	< 5.5
Transverse, radial, 20 cm	5.77 ± 0.04	5.8 ± 0.04	< 7.2
Transverse, tangential, 10 cm	4.33 ± 0.02	4.34 ± 0.03	< 5.5
Transverse, tangential, 20 cm	4.94 ± 0.05	4.89 ± 0.05	< 7.2
Axial, 1 cm	4.06 ± 0.46	4.29 ± 0	< 4.9
Axial, radial, 10 cm	4.29 ± 0.12	4.43 ± 0.10	< 5.5
Axial, radial, 20 cm	4.64 ± 0.07	4.7 ± 0.13	< 7.2
Sensitivity (cps/GBq)			
Centered	6.22	5.21	≥ 5.1
10 cm Off-centered	6.17	5.34	≥ 5.1
Count rates and scatter fractions			
Peak true count rate (kcps)	660 @51.7 kBq/mL	595.59 @51.3 kBq/mL	> 675.000
Peak NECR rate (kcps)	161,8 @51.75 kBq/mL	173.63 @51.35 kBq/mL	> 148.000
Location of NECR peak (kBq/mL)	51.75	51.35	> 25.000
Scatter fraction (%) at low count	31 @0.14 kBq/mL	31,2 @0.14 kBq/mL	
Scatter fraction (%) at NECR peak	33.12	33	≤ 35.00
Max relative count rate error (%)	6.01	5.06	< 12.00
Image quality (%)			
10 mm 4:1	59.9	82.9	> 26
13 mm 4:1	75.4	78.0	> 40
17 mm 4:1	96.6	83.8	> 50
22 mm 4:1	90.3	91.4	> 60
28 mm cold	84.1	79.5	> 68
37 mm cold	85.4	87.1	> 70
Variability (%)			
10 mm 4:1	9.5	8.8	< 11
13 mm 4:1	7.7	7.3	< 9
17 mm 4:1	6.1	5.9	< 8
22 mm 4:1	4.6	4.5	< 8
28 mm cold	3.3	3.5	< 7
37 mm cold	2.2	2.8	< 7
Lung residual	6.5	5.8	≤ 15

Quality image was also evaluated with the percentage contrast for spheres with different diameters, the percentage background variability, and percentage relative lung error for the images. Measured values were compared to recommended values provided by the manufacturer in the Installation Acceptance Test Document

### Sensitivity

**Fig. 2:**
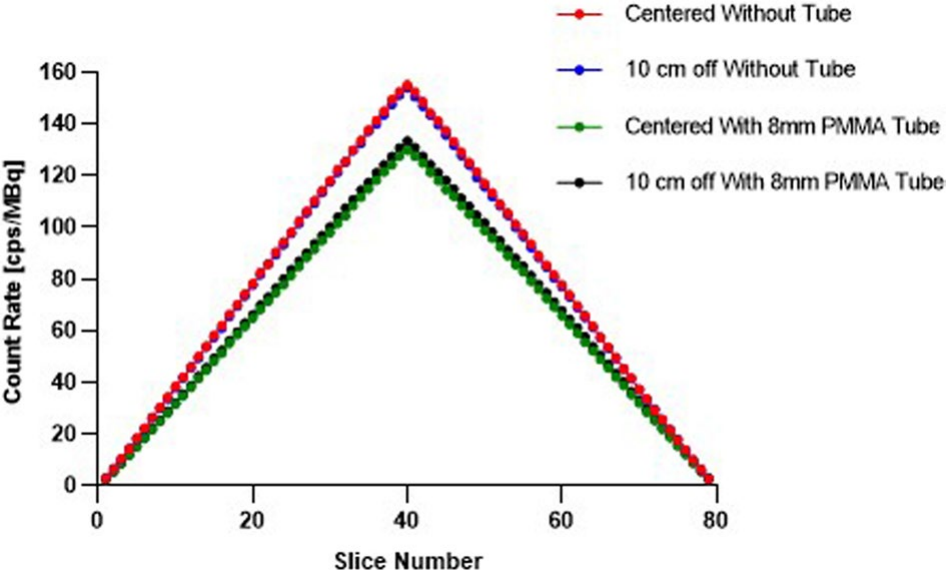
Axial sensitivity profiles for measurements with line source in center of FOV and at 10-cm radial offset with or without 8 mm thickness PMMA tube

The axial sensitivity profiles showed a decrease of the sensitivity with the 8 mm PMMA tube compared with the measurements performed without the tube. The sensitivity was not modified along the PET system when the line source was placed either at the center of the FOV or at 10 cm radial offset (Fig. 2).

The values of sensitivity decreased with the PMMA tube at the center of the FOV from 6.22 cps/GBq to 5.21 cps/GBq (16.2% decrease) and at 10 cm off-centered from 6.17 cps/GBq to 5.34 cps/GBq (13.5% decrease). However, these values were still in accordance with manufacturer prescribed values (Table 2).

### Count rate performance, scatter fraction and accuracies of corrections

**Fig. 3:**
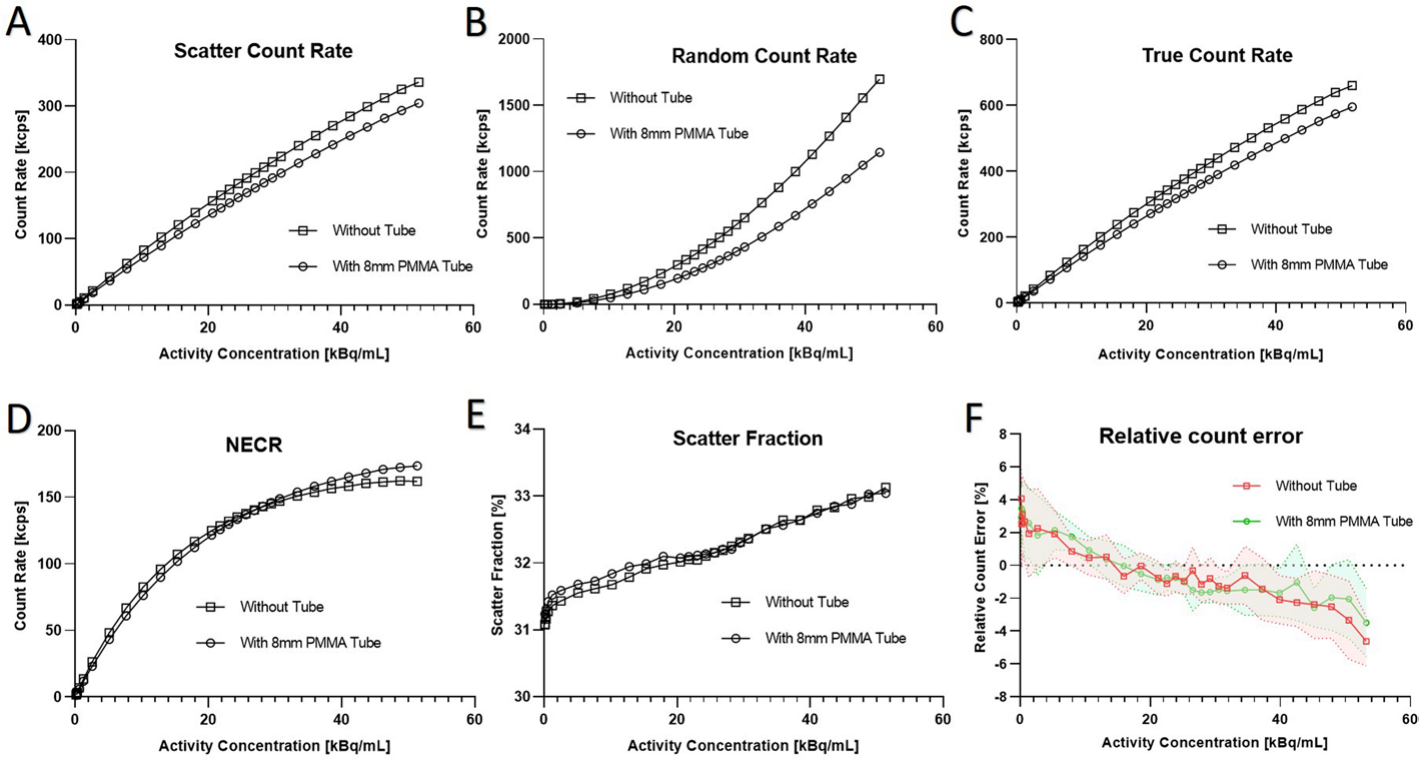
Count rate performance, scatter fraction and count rate accuracy. **A** Scatter Count Rate, **B** Random Count Rate, **C** True Count Rate, **D** NEC rate, **E** Scatter fraction, **F** Relative Count Rate Error: Mean relative count rate errors for the different activity distributions. Mean values are shown as means (full lines) ± maximum/minimum values (dashed lines) with (red) and without (green) the PMMA tube

The presence of the tube slightly decreased the scatter, true and more specifically the random count rates with an enhanced effect for the highest activity concentration (Table 2, Fig. 3). However, those decreases of count rates, mostly reflecting the sensitivity decrease, did not affect significantly the Noise Equivalent Count Rate (NECR) (Fig. 3D) or the scatter fraction percentage (Fig. 3E).

The maximum relative count rate error obtained at low and high count rates decreased from 6.01% without PMMA tube to 5.06% with the 8 mm PMMA tube (Table 2).

These values without and with the 8 mm PMMA tube were within the manufacturer recommended values.

### Image quality phantom

**Fig. 4:**
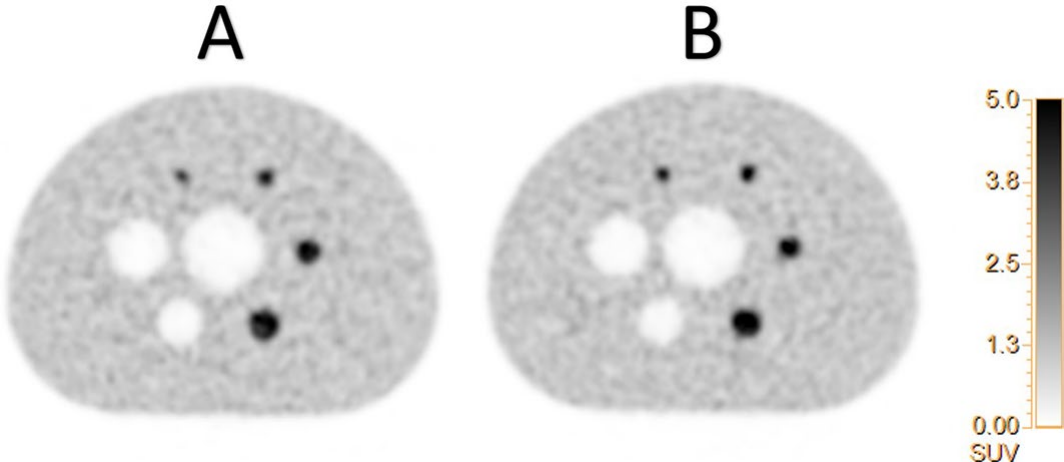
Central slice of the image quality phantom **A** without and **B** with the PMMA tube

For the hot spheres of 13, 17, 22 mm diameter and also for the ‘cold’ ones, the contrast recovery values were similar with or without tube, with all spheres a sphere-to-background ratio of 4:1 (Fig. 4; Table 2, *p* = 0.72, paired t-test). The NECR was identical without or with PMMA tube except for the activity concentration above around 40 kBq/mL (Fig. 3D). The background variability and the average lung residual were not significantly impacted by the presence of the PMMA tube (Table 2, *p* = 0.38, paired t-test), and measurements were in accordance with prescribed manufacturer values and previous published studies [13].

## Discussion

In order to support research on infectious diseases induced by BSL-3 pathogens, we reconfigured a PET/CT imaging system, with minimum of components within BSL-3 laboratory, and we developed an extension of the BSL-3 laboratory into the bore of the machine with the insertion of a PMMA tube, similar to the facilities of another group at the National Institute of Allergy and Infectious Diseases Integrated Research Facility at Fort Detrick, Frederick, Maryland for BSL-4 laboratory [12] (Fig. 1). Among the different materials that could have been used for the tube, the PMMA was chosen over the polycarbonate for its higher resistance required regarding the needed length for the tube and for its easier manufacturing.

In order to validate our PET/CT imaging studies in Non-Human Primates (NHP), the impact of the PMMA tube on the performance of the Vereos-Ingenuity PET/CT device was evaluated according to NEMA NU2-2012 standard.

The thickness of transparent poly-methyl methacrylate (PMMA) constituting the tube, influenced the level of measured noise in CT acceptance test. Indeed, 10 mm thickness PMMA led to an unacceptable level of noise, whereas the introduction of an 8 mm thickness transparent PMMA biocontainment tube into the bore of the imaging system did not drastically modified the performance of the PET/CT system according to the NEMA measurements. With this 8 mm thickness PMMA tube, we observed a slight decrease in sensitivity, although it is completely acceptable according to the prescribed values and comparable with previously published study [13] (Fig. 2, Table 2). Count rate performances were a little diminished for the highest activity concentrations, without affecting the Noise Equivalent Count Rate (NECR) except above 40 kBq/mL, or the scatter fraction percentage (Fig. 3). However, the usual activity concentrations used in our NHP studies were below 20 kBq/mL, with a range of 5 to 15 kBq/mL in the case of [^18^F]-FDG uptake in SARS-CoV-2 infected NHP in lung PET-CT imaging for instance [4]. Within this range of activity concentration, our study showed that count rate performances were not drastically impacted by the presence of the tube. Furthermore, the recent introduction of digital photon counting (DPC, dSiPM) PET/CT system by Philips Healthcare improved the performance of this imaging technology compared to analog PET devices as previously showed [13, 16] by leading to a more precise detection of the small lesions or pathological areas [17] and allowing the injection of less activity in NHP for our PET imaging studies when compared to similar studies [4, 18]. Regarding the peak true count rate values, a lack of initial injected activity for both measurements prevented us to reach the manufacturer recommendations for this parameter but with no impact on other measured parameters during these tests.

Thus the different measured indicators of quality of the images acquired in the phantom experiments were all in the range of recommended values in the presence of the 8 mm PMMA tube.

## Conclusion

The performances of our Vereos digital PET/CT system were evaluated regarding NEMA NU2-2012 standards before and following the reconfiguration of our installation by the introduction of a BSL-3 biocontainment tube directly within the gantry. Modifications of system performances were detected mainly regarding CT noise and PET sensitivity but these performances still remained in the range of recommended standard values. This installation dedicated to the study of human infectious diseases allows a facilitated access in one hand for the maintenance of the PET/CT device in a BSL-1 side and in the other hand, for the veterinary team to the examined animal in BSL-3 side.

## Data Availability

Not applicable.
